# Canopy Density, but Not Bacterial Titers, Predicts Fruit Yield in Huanglongbing-Affected Sweet Orange Trees

**DOI:** 10.3390/plants12020290

**Published:** 2023-01-07

**Authors:** Amit Levy, Taylor Livingston, Chunxia Wang, Diann Achor, Tripti Vashisth

**Affiliations:** 1 Citrus Research and Education Center, University of Florida, Lake Alfred, FL 33850, USA; 2 Department of Plant Pathology, University of Florida, Gainesville, FL 32611, USA; 3 Horticultural Sciences, University of Florida, Gainesville, FL 32611, USA

**Keywords:** citrus, Huanglongbing, light interception, fruit yield

## Abstract

In Florida, almost all citrus trees are affected with Huanglongbing (HLB), caused by *Candidatus* Liberibacter asiaticus (CLas). We characterized various parameters of HLB-affected sweet orange trees in response to yield-improving nutritional treatment, including canopy volume, canopy density and CLas Ct values, and found that the treatment improved yield and maintained canopy density for over three years, whereas untreated HLB-affected trees declined in canopy density. The nutritional treatment did not affect CLas titer or the tree canopy volume suggesting that canopy density is a better indicator of fruit yield. To further validate the importance of canopy density, we evaluated three independent orchards (different in tree age or variety) to identify the specific traits that are correlated with fruit yields. We found that canopy density and fruit detachment force (FDF), were positively correlated with fruit yields in independent trials. Canopy density accurately distinguished between mild and severe trees in three field trials. High and low producing HLB trees had the same Ct values. Ct values did not always agree with CLas number in the phloem, as visualized by transmission electron microscopy. Our work identifies canopy density as an efficient trait to predict yields of HLB-affected trees and suggests canopy health is more relevant for yields than the CLas population.

## 1. Introduction

Huanglongbing (HLB), or citrus greening, has devastated citrus production in Florida and is spreading worldwide. The disease is caused by the Gram-negative, intracellular phloem limited bacteria *Candidatus* liberibacter asiaticus (CLas) and is transmitted by the Asian citrus psyllid (ACP; *Diaphorina citri*). HLB results in bitter and asymmetrical fruit, seed abortion, and premature fruit drop [1]. Other disease symptoms include blotchy mottle (non-symmetrical chlorosis or mottling), pale yellow leaves, yellow shoots, corky veins, stunting, damaged roots, and twig dieback. In leaves and stems, a microscopic phenotype typically associated with CLas infection is the accumulation of callose inside the phloem sieve plate pores [2,3], which begins at early stages of the disease and leads to the collapse of the phloem at more advanced disease stages. As a result, the products of photosynthesis cannot be effectively transported from the leaves, so they are converted into starch and deposited among the grana stacks of the chloroplast [3]. Eventually, the starch deposits become so large that they destroy the grana stacks, rendering the chloroplast inoperative [2].

In Florida, HLB has reduced the production of citrus by 74% [4]. Current management options for HLB are limited and heavily rely on the application of insecticides for controlling the insect vector, *D. citri*. However, insecticide treatment is unsustainable due to the development of resistance among ACP populations, damage to the environment and beneficial organisms, and high cost. Similar challenges and problems also arise when applying antibiotics to control the bacterial population in the trees. Enhanced nutritional treatments, such as controlled release fertilizer (CRF), were shown to increase fruit yield and quality [5], but these treatments can take long time to show impact on yield. There is an ongoing effort to develop HLB resistant or tolerant citrus varieties, but currently there is no known such variety that can be used in the field.

The successful development of HLB therapies require an efficient method to test the effectiveness of the treatments in improving yield and tree health. HLB has spread to all growing areas in Florida, and there are no uninfected control trees. There is a need for a method that can easily determine the impact of treatments on fruit yields. The most common method to determine HLB severity levels is by quantifying CLas with polymerase chain reaction (PCR). This method is used to determine if treatments result in lower amount of CLas in the plants [6,7,8,9]. Regular PCR is used in order to detect CLas DNA in the plant sample, and quantitative PCR (qPCR) is used to measure the number of copies of CLas DNA, corresponding to its titer [10]. With qPCR, the impact of different treatments on HLB is usually determined by the Cycle threshold (Ct) values [11,12], and the relative change in CLas titer in response to treatment is determined with the 2^−ΔΔCt^ method [13]. By including known amounts of plasmid with the corresponding CLas sequence in the qPCR analysis, the number of cells of CLas per gram of plant tissue sample can be also predicted [14]. These values are then used to quantify the relative change in CLas population in response to treatment [6]. Other techniques include microscopic, spectroscopic, and imaging techniques, but those are more complex and expensive [15]. In the field, HLB disease severity levels are determined by measuring Disease Index Rating [7,16], however it’s a non-standardized, subjective rating method.

For growers, the most important and relevant value for scoring HLB mitigation treatments is the effect on the fruit yield of the tree. However, it can take multiple years to see an effect of treatment on the yield [17], obtaining this value is very laborious, and it can only be done at certain times of the year. Moreover, in many cases, there is a need to determine the effect of treatments while trees are still in their juvenile state, for example when analyzing genetically edited or modified plants. Therefore, finding an easier and reliable method to predict fruit yield, can help the assessment of HLB therapies.

Here, we conducted series of field experiments to identify most important attribute of HLB trees in the field with the goal of determining which of these values correlates with the yield. Recently it has been documented that enhanced mineral nutrition treatments can improve productivity of HLB-affected trees [5,18]. Therefore, we conducted a small-scale field trial to determine which parameters of HLB-affected trees change along with fruit yield in response to fertilizer treatments. Altogether, our results show that fruit detachment force (FDF), and canopy density can be used to determine the effect of HLB on tree performance under field conditions. Our results suggest that canopy health is more relevant to yield than CLas population size as routinely determined by qPCR method.

## 2. Results

### 2.1. Canopy Density, but Not Canopy Volume or qPCR Values, Are Related to Yield Increase

**Figure 1:**
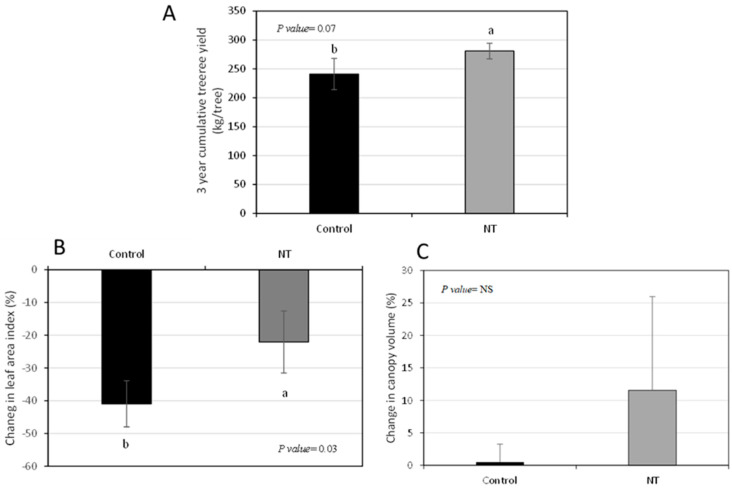
Changes in yields and canopy density and volume after CRF treatment. After three years of CRF treatment, trees had higher yields (**A**). Loss of canopy density (measured as leaf area index) was smaller in CRF treated trees than in controls (**B**). There was no difference in canopy volume (**C**).

In order to identify parameters that are associated with improvement in tree health and yields, we analyzed trees treated with control release fertilizers with enhanced nutrients treatment, which provides a constant and balanced supply of nutrients and has been shown to significantly improve yields. We employed these phenomena to identify parameters that are associated with improvement in tree health and yields. HLB trees were treated for 3 years with CRF plus 50% higher rate of Mn and B and we analyzed the changes in the yields, canopy volume, canopy density (LAI) and in CLas levels during this time. After three years, the enhanced nutrition treatment increased the tree yields by about 20% (Figure 1A) compared to control. There was a difference in the changes of the leaf area index during the experiment between the untreated and treated trees- the canopy of the trees that received the enhanced nutrition remained denser than the canopy of the control trees (Figure 1B). There was no difference in how the canopy volume changed during the trial between the treated and untreated trees (Figure 1C). Surprisingly, although enhanced nutrition treatment led to increase in yield and canopy density, there was never any difference in the bacteria levels in the trees between treated and untreated trees throughout the four years of the experiment, based on qPCR results (Table 1). These results suggested that higher yields in HLB trees are associated with higher canopy densities, but not with higher canopy volumes or lower CLas levels.

### 2.2. Evaluation of Trees According to Light Interception (%INT)

**Figure 2:**
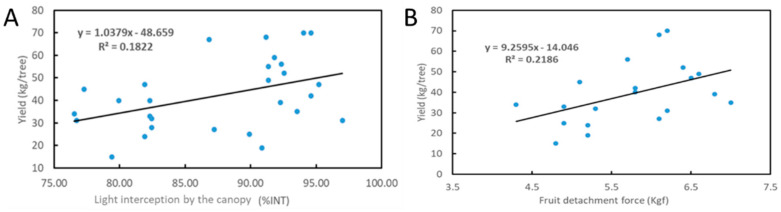
Correlation between yield and photosynthetically active radiation interception percent in the canopy (%INT; (**A**)) or fruit detachment force (**B**).

Next, we tested if canopy density, can be used by citrus growers as an accurate measurement to assign disease severity for HLB trees. We tested trees in three different blocks—young Valencia trees (11 years old), old Valencia trees (18 years old) and old Hamlin trees (19 years old). Trees were divided into two categories based on %INT levels, where trees with an %INT of 85 and over were categorized as mild HLB, and those below 85 were categorized as severe HLB (there are no HLB negative trees in Florida). We measured the canopy volumes, disease indexes, root densities, SPAD, %INT, fruit sizes, FDF, yields, CLas Ct values, and CLas numbers of the trees in each block. In all the three field trials, trees that were categorized as ‘severe HLB’ according to the %INT had around half of the yield levels, compared to those categorized as the ‘mild HLB’ category (Table 2). The severe HLB trees also had smaller fruit and lower FDFs than mild trees. Correlation analysis of the results show that %INT and FDF are both correlated with the yields (Figure 2). There was no difference in canopy volumes, disease indexes, root densities and SPAD values between the severe and mild HLB trees (Table 2). We also performed the measurement of macronutrients, which include nitrogen (N), phosphorus (P), potassium (K), calcium (Ca), sulfur (S), magnesium (Mg) and micronutrients, which include boron (B), zinc (Zn), manganese (Mn), iron (Fe), copper (Cu). The results showed there were no significant differences among the trees in the fields (Supplemental Table S1). Remarkably, there was also no difference in amount of CLas cells per gram tissue, as determined by qPCR Ct value (Table 2). These results indicate that %INT (canopy density), but not Ct values, can be used accurately to determine HLB disease levels and predict yields.

### 2.3. qPCR May Not Always Accurately Detect Live CLas Cells

**Figure 3:**
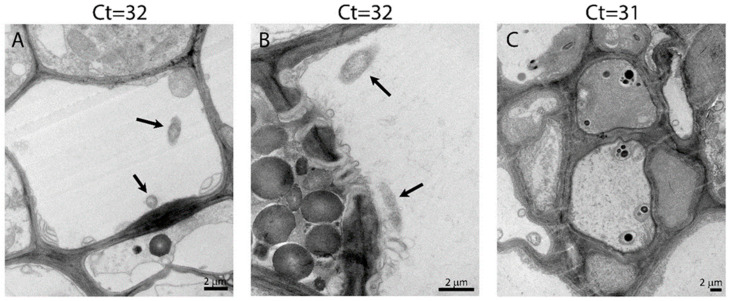
CLas cells in Cmac phloem. CLas bacterial cells were found in midrib sieve elements of a plant with Ct values of 32 (**A**,**B**), but no cells were found in midrib of plant with Ct value of 31 (**C**). CLas cells are marked by an arrow.

To further test if Ct value is a reliable measure for number of bacteria, we used quantitative PCR and transmission electron microscopy (TEM) in order to visualize CLas in *Citrus macrophylla* (Cmac) and grapefruit plants in greenhouse that were infected with CLas. qPCR and TEM analysis were conducted on the same leaf midribs and results were compared (Table 3). In grapefruit, five months after infection by ACP, in plants that had ‘negative’ Ct values (Ct value above 32) we could not find any CLas cells in our TEM analysis, but we could find few bacterial cells (2 cells in 31 sieve elements examines) in the plant that had a ‘positive’ Ct value (Ct value below 32). In contrast, in Cmac, we detected the highest number of CLas cells (8 CLas cells in 10 sieve elements examined) in the phloem of a plant that was ‘negative’ according to the Ct value. In the midrib that had positive Ct value, we could not detect any CLas cells in any of the sieve elements examined (Table 3, Figure 3). These results suggest that qPCR may not always reliably detect CLas cells, since that the sensitivity of the analysis is not optimal to deal with the low levels of CLas.

## 3. Discussion

Accurate evaluation of the HLB disease status is of major importance to test the efficiency of treatments, especially in field settings [6]. Predicting the number of CLas cells per gram plant tissue is one of the most common ways of estimating HLB disease status. However, the correlation between lower CLas Ct values and lower yields was not proven. Here, CLas Ct values and the numbers of CLas cells were correlated with fruit yield in four independent field trials, but no significant correlation was found. These results indicate that Ct value, at least as it is determined today, is not a good predictor of HLB disease severity. The lack of correlation may result from a lack of sensitivity of the qPCR method, especially as CLas is phloem localized and its levels are very low in infected citrus trees [19]. In addition, it was shown that CLas does not distribute equally in the tree [20], and therefore sampling can affect the quantification. In this work, we attempted to overcome this limitation by sampling many leaves that looked similar in appearance and age (30 leaves per tree) per tree. However, we cannot rule out that the sampling influenced the results. Another possible explanation may be that qPCR is detecting dead, as well as living, bacteria. In fact, Etxeberria et al. [21] measured CLas qPCR Ct values in leaves of ‘Valencia’ orange trees after a heat-treatment that eliminated viable CLas, and found that although titer declined, CLas remained detectable 5 months after treatment, supporting this idea. Here, we showed that we could find viable CLas in leaves of Cmac that had a Ct that is considered as negative (above 32), suggesting an even more complex situation. Previous work showed very low levels of CLas were present in vegetative flush tissues of HLB-affected plants [22]. In that work, it was shown that although no CLas were detected in these leaves, the host plants still responded to the bacterial infection, and the phloem were plugged with callose. Thus, it is also possible that HLB disease is not directly correlated by the number of bacteria in the stem, and the host plant responses may play a significant role in disease development. Further work is needed to understand the efficiency of qPCR in determining CLas levels.

We analyzed different possible field measurements for yields in field settings and identified two values that were positively correlated with the yield: FDF and %INT. These values are all important parameters in the study of yield estimates in horticulture and are all known to be related to HLB symptomology and are therefore expected to serve as reliable indicators to assess effects therapeutic treatments on diseased plants. For example, it was already shown that HLB-affected trees display higher premature fruit drop, and lower FDF [23,24]. Comparing susceptible HLB varieties to tolerant ones also showed that lower drop rate is associated with HLB tolerance [25]. In HLB-tolerant varieties, internal structures were better preserved compared to susceptible ones [26]. It was suggested that CLas effector SDE1 is inducing senescence in citrus, thus providing a possible link between the bacteria and the fruit drop phenomena [27].

Dividing disease severity of trees according to %INT in three independent field trials (with different varieties and tree ages) into severe or mild, resulted in clear low and high yield categories, respectively. Intercepted PAR (%INT) indicates the amount of PAR caught by various canopy layers as the PAR travels through the canopy. % INT was measured above an inside the canopy and represents the amount of light absorbed by the leaves (transmissibility). The %INT value is represented as percentage (of PAR inside the canopy compared to the PAR at the edge), such that higher %INT indicates higher canopy density. Using the %INT has the advantage of being a relative rather than absolute measurement. Therefore, %INT measurement is not dependent on the absolute size of the canopy and could be appropriate for field settings.

Our study shows that evaluating potential tree productivity (yield) according to CLas Ct values is inaccurate. It is still not clear if the lack of correlation between Ct values and yields results from a problem with PCR sensitivity, or uneven distribution of CLas in the tree (making sampling difficult), or that the development of HLB does not depend on the level of the bacteria. This lack of connection may indicate that treatments aimed at lowering the bacteria levels in the trees may not have a significant effect on HLB.

Overall, our results show that fruit yield in HLB-affected trees is directly correlated with the canopy density (trees with higher yields have higher density), and with fruit FDF and size (trees with higher yields have larger fruits with higher FDF). Employing these measurements with trees in the field can be used to evaluate HLB severity. The importance of canopy volume in HLB disease was shown previously in Brazil [28]. Here, we suggest that canopy density may provide a more accurate and holistic determination of tree health status. Scoring tree health status with ‘disease index rating’ can similarly estimate the status the canopy, but this measurement depends on subjective estimation of the canopy symptoms. %INT can provide an easy and objective way to quantify the density of the canopy using simple and cheap instruments. Growers can use %INT to evaluate and compare trees. Overall, our results indicate that alongside controlling the bacteria, treatments to keep the canopy vigorous and the normal development of the fruit (for example, by enhanced nutrition or hormonal treatments) will have a positive effect on yields and should get more attention.

## 4. Materials and Methods

### 4.1. Plant Materials and Experimental Design

Three distinct experimental orchards at the University of Florida Citrus Research and Education Center in Lake Alfred were used for this research. Orchard 1 and 2 included eighteen-year-old and eleven-year-old ‘Valencia’ sweet orange trees on Swingle citrumelo (*Citrus paradisi* × *Poncirus trifoliata*) rootstock, respectively; orchard 3 included nineteen-year-old ‘Hamlin’ sweet orange trees on Swingle citrumelo. These trees were selected to represent different ages and varieties of sweet orange, which is the most important citrus crop in Florida. Currently, because of the prevalence of HLB in Florida, it is unlikely to find healthy CLas-negative sweet orange trees as controls in open-air groves. Therefore, trees (at least 5 single tree replicates were used for each orchard) exhibiting mild and severe visual symptoms of HLB were selected. The common tree health indicators (CLas titer, canopy volume, leaf area index (LAI), photosynthetically active radiation (PAR) interception percent in the canopy (%INT), root density, leaf nutrient, leaf SPAD index, disease index rating, fruit size, fruit detachment force (FDF), and yield) were collected on individual trees on the day of fruit harvest. Comparing transmission electron microscopy (TEM) and Ct values was performed on 5-month-old CLas-positive seedlings of Cmac and ‘Duncan’ grapefruit (*C. paradisi*) grown under greenhouse conditions at the Citrus Research and Education Center (Lake Alfred, FL).

For the enhanced nutrition treatment experiment, twelve-year-old ‘Valencia’ sweet orange trees on Swingle citrumelo rootstock grown in commercial orchard at Fort Meade, Florida were used. Trees were regularly irrigated (via microsprinklers) and grown under standard commercial grove management practices, which included regular insect and disease control. The site was on the Mid-Florida Ridge and soil pH of the grove was identified in the ideal range, ranging from 6.5 to 7.2. Based on recent findings about effect of nutritional treatments and soil acidification on improving performance of HLB-affected trees [5,9,29,30,31], an enhanced nutritional treatment was compared to control trees. The control trees were fertilized according to standard University of Florida, Institute of Food and Agricultural Sciences (UF IFAS) guidelines for citrus fertilization. Control trees received macro and secondary nutrients via soil application of granular fertilizer (which quickly releases nutrients) and micronutrients, zinc, manganese, iron, and boron were applied as foliar sprays. Enhanced nutritional treatment received macro and secondary nutrients as a controlled release fertilizer (CRF) and micronutrients were also soil applied micronutrient. Both control and the enhanced nutritional treatment were applied 3 times a year. The rates of each nutrient applied for both treatments is provide in supplemental Table S1. The treatments were evaluated for CLas titer, canopy volume, canopy density, and individual tree yield as per the method described below.

### 4.2. Genomic DNA Extraction and Ca. L. asiaticus Titers

Thirty random, fully mature leaves, free of any defect were collected. The midribs of these leaves were excised using a clean razor blade and stored at −80 °C until DNA extraction. Samples of the leaf tissue midrib (100 mg) were grinded by TissueLyser II (Qiagen, Germantown, MD 20874, USA) using liquid nitrogen, extracted in 0.5 mL of extraction buffer (100 mM Tris-HCL, pH 8.0; 50 mM EDTA; 500 mM NaCl; 2% CTAB, and 2% PVP 40). The extract was incubated at 65 °C for 30 min. Following incubation, 500 μL of 5 M potassium acetate was added, mixed thoroughly, and incubated on ice for 20 min. The mixture was centrifuged at 13,000 rpm for 10 min, 400 μL of supernatant was recovered, and DNA was precipitated by adding an equal volume of isopropanol and held at −20 °C overnight. The DNA was pelleted, washed, and resuspended in 100 μL of water for PCR analysis. qPCR was performed with primers and probes (HLBas, HLBr, and HLBp) for the ‘Ca. L. asiaticus’ bacterium [32] using ABI PRISM 7500 sequence detection system (Applied Biosystems, Foster City, CA) in a 10-μL reaction volume consisting of the following reagents: 200 nM (each) target primer (HLBas and HLBr), 100 nM target probe (HLBp), and 1 × TaqMan qPCR Mix (Applied Biosystems). The amplification protocol was 94 °C for 5 min followed by 40 cycles at 94 °C for 10 s and 58 °C for 40 s. All reactions were performed in triplicate and each run contained one negative (DNA from healthy plant) and one positive (DNA from ‘Ca. L. asiaticus’-infected plant) control. Data were analyzed using the ABI 7500 Fast Real-Time PCR System with SDS software. The resulting cycle threshold (Ct) values were converted to the estimated bacterial titers using the grand universal equation

(1) Y=13.82−0.2866X

where *Y* is the estimated log concentration of templates and *X* is the qPCR Ct values, as described by Li et al. [33].

### 4.3. Disease Index Rating

Each tree was rated based on the disease index rating system developed by the Citrus Research and Development Foundation in Lake Alfred, FL [34]. Briefly, each side of the tree was divided in quarters and was given a score out of 5 based on the HLB symptoms, where 0 means highly symptomatic and 5 means no HLB symptoms. All the scores were summed up to give the tree a total score out of 40.

### 4.4. Canopy Measurements

Canopy volume was measured at time of harvest for each tree individually. Canopy volume, expressed as cubic meters was calculated using a geometric prolate spheroid Formula (2):

[(4/3) (π) (H/2) (ACR)2](2)

where π = 3.14, H = tree height, and ACR = average canopy radius. ACR was calculated by dividing tree diameter by 2 and calculating average radius. Tree diameter was measured in two directions: east to west (D1) and north to south (D2).

HLB-affected trees often have sparse canopies; therefore, canopy density can be a good indicator of tree health and growth. Leaf area index (LAI) was measured for each tree to estimate canopy density. A plant canopy imager (CI-110; CID Bio-Science, Camas, WA) was used to measure the LAI and all the measurements were taken in the morning of a sunny day at the center of the canopy. The instrument was equipped with numerous light sensors to measure photosynthetically active radiation and with a global positioning unit to calculate the zenith angle for an accurate measurement of LAI. In addition to LAI, PAR interception (%INT) by the canopy was measured to provide another estimation of canopy density. %INT determines the percentage of PAR that is intercepted by the canopy, and is an [35,36], easy, and quick alternative method to measure canopy density; “the higher the %INT, the denser the canopy is and vice versa”. For %INT, the PAR was measured outside the canopy and inside the canopy (average of the PAR in each quarter of the canopy). The %INT was calculated by (3):

[(PARoutside − PARinside)/PARoutside] ∗ 100(3)

### 4.5. Root Density

Root density was measured at the time of harvest. From each tree, eight evenly spaced soil cores, ≈1 m from the trunk of the tree, were taken from the wetted zone. Roots were sifted, washed, and dried in an oven at 50 °C for 48 h, then the dry weight of the root samples was obtained. Root density was expressed as milligram of dry weight per cubic centimeter of soil.

### 4.6. Leaf Nutrient Analysis

Thirty random leaves with intact petioles per tree were collected from nonfruiting branches. The collected leaves were washed using acidic soap, then the leaves were dried for 48 h in a convection oven (Thermo Fisher Scientific, Waltham, MA, USA) and ground to a fine powder. Ground leaves were sent to Waters Agricultural Laboratories (Camilla, GA, USA) to perform a standard leaf nutrient analysis [29].

### 4.7. SPAD Index

The soil plant analysis development (SPAD) index was used to estimate the total leaf chlorophyll content of thirty fully expanded, randomly selected, mature leaves per tree using a chlorophyll meter (MC-100 Chlorophyll Concentration Meter; Apogee Instruments, Logan, UT, USA). The thirty SPAD values of each tree were averaged for a representation of the chlorophyll content of the tree.

### 4.8. Yield, Fruit Size and Fruit Detachment Force (FDF)

When the total soluble solids to titratable acidity ratio of fruit reached commercial harvest standards, the fruit were hand harvested by commercially trained harvesters. ‘Valencia’ trees in orchards 1 and 2 were harvested on 3 and 12 March 2020, respectively and ‘Hamlin’ were harvested on 4 December 2019. Fruit yield is expressed as total kg of fruit per tree. For each tree, at time of harvest, 20 fruit attached to the tree branches were collected by clipping the branches. Only the branches bearing single fruit at the distal position were collected. All 20 fruits were used for fruit size measurement using a handheld digital caliper to measure the equatorial length on all 20 fruits. For each fruit, FDF was measured using a digital force gauge (Force One; Wagner Instruments, Greenwich, CT). FDF determines how much force is required for the fruit to be “pulled” or detached from the tree/branch. FDF > 6 kgf [37] is considered as a fruit that is not physiologically ready to abscise, FDF ≤ 6 kgf represents a fruit that has high tendency to drop at the time of collection.

### 4.9. HLB Inoculation for TEM

The *Diaphorina citri* colony infected with HLB was maintained inside an insect cage (Nasco Scientific) on Cmac that is also infected with HLB. These infections were confirmed through qPCR. The *D. citri* colony was reared at 25 ± 2 °C under a 14 light:10 h dark photoperiod and 60–70% relative humidity. The colony is tested monthly by qPCR for infection rate. Citrus seedlings, (6 months to a year old) that had young flush were placed inside a separate insect cage in the same room with the same conditions. Twenty-five to fifty individual psyllids from the infected colony were collected with an insect aspirator (Carolina Biologic) and transferred to the cage containing the seedlings. Seedlings were tested monthly for HLB with qPCR after 4–8 weeks. At 8 weeks, seedlings were trimmed, treated with M-pede/Malathion insecticide, and relocated to a holding area for 2 weeks where they were monitored for psyllid activity. Once it had been confirmed that the seedlings were psyllid free and HLB positive, they were placed in a temperature-controlled greenhouse.

### 4.10. Transmission Electron Microscopy (TEM)

To compare CLas seen in TEM with Ct values, one half of a leaf midrib was subjected to CLas detection with qPCR, as described above, and the other half of the same midrib was subjected to TEM analysis to identify CLas in the phloem, performed as described by Achor et al. [22]. Briefly, Samples were fixed with 3% (*v*/*v*) glutaraldehyde in 0.1 M of potassium phosphate buffer at pH 7.2 for 4 h at room temperature, washed in phosphate buffer, then postfixed in 2% osmium tetroxide (*w*/*v*) in the same buffer for 4 h at room temperature. The samples were further washed in the phosphate buffer, dehydrated in a 10% acetone (*v*/*v*) series (10 min per step), and infiltrated and embedded in Spurr’s resin over 3 days. Sections (100-nm) were mounted on 200-mesh formvar-coated copper grids, stained with 2% aq uranyl acetate (*w*/*v*) and Reynolds lead citrate, and examined with a Morgagni 268 transmission electron microscope (FEI).

## Figures and Tables

**Table 1:** CLas Ct values at the beginning of CRF treatments (2016) and during the three years of CRF experiment.

Treatment	Block	2016	2017	2018	2019
Control	1	26.40	35.46	25.41	25.13
Control	2	25.98	32.77	31.56	26.89
Control	3	29.84	30.06	27.66	29.01
Enhanced Nutritional Treatment	1	28.01	31.26	32.01	27.41
Enhanced Nutritional Treatment	2	26.49	31.70	32.63	28.46
Enhanced Nutritional Treatment	3	26.87	29.13	35.73	26.30
	*p* value	0.85	0.2	0.13	0.83

**Table 2:** Characterizing HLB trees according to %INT.

Tree Description	Visual Symptoms	Canopy m^3^	DI	Root Den.	SPAD	%INT	Fruit Size	FDF	Yield kg/tree	Ct	CLas Cells/gram
11-year old Valencia trees	Severe	6.63 ± 0.83	25.17 ± 3.04	0.68 ± 0.14	61.03 ± 1.35	85.28 ± 1.91	63.79 ± 0.8	5.25 ± 0.18	23.67 ± 2.4	30.03 ± 0.4	4.87 × 10^9^ ± 1.17 × 10^9^
	Mild	7.09 ± 1.08	20.83 ± 1.37	0.85 ± 1.17	58.72 ± 0.77	94.18 ± 0.72	65.78 ± 0.6	6.45 ± 0.17	41.00 ± 3.14	29.27 ± 0.3	7.82 × 10^9^ ± 1.81 × 10^9^
	*p* value	ns	ns	ns	ns	0.0008	0.0229	0.015	0.0056	ns	ns
18-year old Valencia trees	Severe	22.97 ± 1.84	34.75 ± 2	0.56 ± 0.06	51.11 ± 4.08	80.83 ± 1.38	61.50 ± 0.85		36.50 ± 4.33	30.81 ± 0.18	2.50 × 10^9^ ± 2.84 × 10^8^
	Mild	24.64 ± 2.1	27.50 ± 0.86	0.64 ± 0.19	57.52 ± 2.5	91.14 ± 1.6	64.13 ± 1		62.75 ± 3.47	30.86 ± 0.08	2.37 × 10^9^ ± 1.48 × 10^8^
	*p* value	ns	0.0662	ns	ns	0.0005	0.0484		0.0435	ns	ns
19-year old Hamlin trees	Severe	20.04 ± 0.74	30.00 ± 4.5	0.40 ± 0.12	59.00 ± 1.9	79.01 ± 1.3	57.43 ± 0.93	5.59 ± 0.3	38.00 ± 2.8	29.72 ± 0.6	8.34 × 10^9^ ± 2.32 × 10^9^
	Mild	18.37 ± 2.3	22.00 ± 1.3	0.70 ± 1.14	57.99 ± 1.76	92.23 ± 0.65	61.50 ± 0.44	6.15 ± 0.18	60.75 ± 4.98	30.20 ± 0.42	4.15 × 10^9^ ± 1.29 × 10^9^
	*p* value	ns	ns	ns	ns	0.0003	0.0402	0.075	0.0613	ns	ns

**Table 3:** Comparison between *Candidatus* Liberibacter asiaticus quantification by qPCR and TEM.

Variety	Plant	Ct Value ^1^	SE ^2^	CLas Cells ^3^
*C. mac*	1	32.09	10	8
*C. mac*	2	31.2	25	0
*C. mac*	3	32.75	14	0
Grapefruit	1	34.9	45	0
Grapefruit	2	34.7	31	0
Grapefruit	3	28.5	31	2

^1^ Ct value < 32 is considered positive. ^2^ SE = Standard Error. ^3^ CLas cells visualized by the TEM in the same phloem tissue.

## Data Availability

Data used for analysis can be obtained through the corresponding author, Tripti Vashisth (tvashisth@ufl.edu).

## Supplementary Materials

The following supporting information can be downloaded at: https://www.mdpi.com/article/10.3390/plants12020290/s1, Table S1: Rate of nutrients applied (kg/hectare) in control and enhanced nutritional treatment.
