# Supplementary material for: Canopy Density, but Not Bacterial Titers, Predicts Fruit Yield in Huanglongbing-Affected Sweet Orange Trees
Source: Plants (Basel). 2023 Jan 7;12(2):290. doi: 10.3390/plants12020290 (PMC9863558; doi:10.3390/plants12020290)
Supplement: Supplementary file 1 [file plants-12-00290-s001.zip › plants-2019893-supplementary.pdf]

Supplemental Table S1: Rate of nutrients applied (kg/hectare) in control and enhanced nutritional treatment.

	**Control**	**Enhanced nutritional treatment**
Nitrogen	262	192
Phosphorus	87.4	64.1
Potassium	349.4	256.3
Calcium	90.6	66.5
Magnesium	48.0	35.2
Zinc	6.7	13.1
Manganese	6.7	13.4
Iron	5.6	6.6
Boron	0.6	2.6
